# Advances in Genetical Genomics of Plants

**DOI:** 10.2174/138920209789503914

**Published:** 2009-12

**Authors:** R.V.L. Joosen, W. Ligterink, H.W.M. Hilhorst, J.J.B. Keurentjes

**Affiliations:** 1 Laboratory of Plant Physiology, Wageningen University, Droevendaalsesteeg 1, NL-6708 PB Wageningen, The Netherlands; 2 Laboratory of Genetics, Wageningen University, Droevendaalsesteeg 1, NL-6708 PB Wageningen, The Netherlands; 3 Centre for Biosystems Genomics, Droevendaalsesteeg 1, NL-6708 PB Wageningen, The Netherlands

**Keywords:** Genetical genomics, e-QTL, network reconstruction, Arabidopsis thaliana, crop genetics.

## Abstract

Natural variation provides a valuable resource to study the genetic regulation of quantitative traits. In quantitative trait locus (QTL) analyses this variation, captured in segregating mapping populations, is used to identify the genomic regions affecting these traits. The identification of the causal genes underlying QTLs is a major challenge for which the detection of gene expression differences is of major importance. By combining genetics with large scale expression profiling (*i.e.* genetical genomics), resulting in expression QTLs (eQTLs), great progress can be made in connecting phenotypic variation to genotypic diversity. In this review we discuss examples from human, mouse, *Drosophila*, yeast and plant research to illustrate the advances in genetical genomics, with a focus on understanding the regulatory mechanisms underlying natural variation. With their tolerance to inbreeding, short generation time and ease to generate large families, plants are ideal subjects to test new concepts in genetics. The comprehensive resources which are available for *Arabidopsis* make it a favorite model plant but genetical genomics also found its way to important crop species like rice, barley and wheat. We discuss eQTL profiling with respect to *cis* and *trans* regulation and show how combined studies with other ‘omics’ technologies, such as metabolomics and proteomics may further augment current information on transcriptional, translational and metabolomic signaling pathways and enable reconstruction of detailed regulatory networks. The fast developments in the ‘omics’ area will offer great potential for genetical genomics to elucidate the genotype-phenotype relationships for both fundamental and applied research.

## 1. INTRODUCTION

Ever since the current paradigm of gene transcription preceding biological function, research on gene function has focused on expression studies. With the ever increasing availability of genomic sequences and the introduction of microarray technology, enabling the high-throughput analysis of gene expression, this has rapidly become a favorite tool for many researchers [1]. In a typical microarray experiment specific conditions or developmental stages are studied by comparing expression profiles and determining differences in gene transcription. The object of profiling can be a single genotype showing phenotypic diversity in a spatial and temporal manner, *e.g.* in different tissues and developmental stages, or when exposed to different growing conditions (*e.g.* [2]). In this way, large compendia of expression data have been acquired, providing ontological information of genes involved in developmental control and environmental responses [3-5].

Although much of the relationship between the temporal expression of genes and their function can be learned from these analyses, often no information can be obtained about the genetic regulation of transcription or whether expression differences are causal for or a consequence of phenotypic differences. Therefore, instead of comparing different conditions of a single genotype, equivalent samples of different genotypes varying in the trait of interest are often analyzed. These can be natural variants within a species, artificial mutants or transgenics like knockout and over expression lines. Such analyses have shown to be extremely powerful in determining directionality in biological pathways, especially in qualitative traits [6, 7]. However, it becomes extremely difficult to define a proper experimental setup when the trait of interest is complex and has a quantitative character. Geneticists are used to deal with this type of complex traits by using the power of natural variation within species.

In segregating populations, derived from crosses between distinct parents and genotyped with molecular markers, linkage is sought between variation in the trait of interest and genotypic diversity [8]. For this purpose a broad range of software tools and statistical analyses are available. The identified genomic regions explaining the observed phenotypic variation are commonly referred to as quantitative trait loci (QTLs), which can subsequently be used for marker assisted breeding purposes without further knowledge of the underlying genes [9]. Whenever the purpose is to identify the causal genes or even nucleotide polymorphisms (QTNs) underpinning a given QTL, one needs to invest in follow-up analyses for fine mapping and ultimately the cloning of a QTL [10]. This approach however, is very labor intensive and time consuming and confines the classical QTL mapping to a low throughput technique. 

Like for many physiological traits, variation in gene expression often shows a quantitative distribution, hence, all the classical statistical tools and concepts for QTL mapping can be applied for its genetic dissection. Thus, subjecting expression variation to linkage analysis identifies genetic regulatory loci, and ideally genes, explaining the observed variation. Knowing the position of genes and their corresponding expression QTLs (eQTLs) renders great opportunities for dissecting quantitative traits. This was first recognized by Jansen and Nap [11] who outlined a concept, coined ‘genetical genomics’, in which the combination of a genotyped segregating population (*i.e.* genetics) and genome-wide expression profiling (*i.e.* genomics) is used to formulate hypothetic regulatory pathways and unravel complex traits in a more high-throughput manner. Analogously, similar approaches can be followed for data derived from other ‘omic’ technologies such as proteomics (pQTLs) and metabolomics (mQTLs) [12].

The first study reporting a proof of principle of genetical genomics was performed in *Saccharomyces cerevisiae* [13]. In a relatively small population of 40 haploid segregants from a cross between a laboratory and a wild type strain, it was shown that parental differences in gene expression were highly heritable and amenable to genetic mapping. This first report was quickly followed by more comprehensive eQTL studies in higher eukaryotes [14] and has now been applied in a broad range of taxonomic kingdoms including yeast [13, 15-17], nematodes [18], insects [19, 20], plants [21-24], rodents [25-27] and humans [28-33]. All studies demonstrated the power of combining gene expression and genetic analyses to refine molecular pathways involved in complex phenotypes and to identify key driver genes thereof. Moreover, they have shown general and conserved mechanisms of expression regulation which improved our understanding of adaptive strategies and evolutionary concepts [19, 34].

In this review we will discuss the genetic architecture of gene expression regulation, embarking on recent findings in the reference plant *Arabidopsis thaliana*, the implications of genetical genomics approaches for crop species and the impact of genetic analyses of ‘omics’ data on the construction of regulatory networks. We will discuss future prospects and speculate on the utilization of advancing technological developments for genetic studies.

## 2. GENETIC ARCHITECTURE OF GENE EXPRESSION VARIATION

The detection of eQTLs depends on a number of factors, which together determine the proportion of genetically regulated genes that can be observed. First, biological factors such as the assayed tissue, developmental stage or environmental conditions and the genotypic diversity present in the mapping population determine which genes are expressed and exhibit allelic variants, respectively. Second, statistical issues like population type and size, genetic map quality, measurement accuracy and the number of genes analyzed determine mapping power and detection thresholds. Because all these aspects vary between different experiments, reported fractions of regulated genes range from only a handful to over 50% of the total gene content.

### 2.1. Regulation *in cis*

Given the prerequisite of allelic variation, there can be many reasons why genes are differentially expressed in genotypically diverse individuals of a species. Well-known phenomena are allelic variants of transcription factors and other regulators, *cis-*elemental variation in promoter sequences, differences in mRNA stability, copy number variation and genomic rearrangements such as translocations, insertions and deletions. The latter include gene loss and duplication, resulting in neo- and sub-functionalization. Most of these variations in DNA structure will result in eQTLs but depending on the position of the causal polymorphism, an important dissection is made in local and distant eQTLs Fig. (**1**) [35]. Local eQTLs can be the result of closely linked *trans*-acting factors but in the majority of cases result from *cis*-regulatory variation in the genes under study. By definition eQTLs acting *in* *cis* affect transcription initiation, rate and/or transcript stability in an allele-specific manner. In addition, *cis*-regulated genes might encode regulators affecting the expression of downstream target genes *in* *trans*. Although the exact proportion varies between studies the occurrence of *cis*-acting eQTLs is substantial ranging from one-third to half of the total number of eQTLs [36].

However, because of limitations in mapping resolution, eQTL support intervals may still contain multiple genes and as a result the classification of *cis*-eQTLs should be used with care. To discriminate true *cis-*regulatory polymorphisms from local *trans-*regulation, allele specific expression (ASE) assays can be performed [37]. In such assays a transcribed polymorphism is used to enable discrimination between the parental transcripts and test for allele specific expression in an F_1_ hybrid. Because both parental alleles share the same genetic background in F_1_ hybrids, and therefore are equally exposed to *trans*-acting factors, any difference in expression can only be explained by true *cis*-acting variation. Usually, ASE-assays are performed by single gene qRT-PCR approaches but the recent development of whole genome SNP-tile microarrays (*e.g.* in Arabidopsis) enables the simultaneous testing of genome-wide ASE [38].

Although expression differences are treated as quantitative traits in mapping approaches, qualitative differences, characterized by a total lack of expression for one of the allelic variants, can also be observed. The variation in a measurable detection signal can be due to differences in hybridization efficiency, which can be confirmed with genomic DNA hybridization, or genuine loss of transcription. Hybridization efficiency differences are often caused by polymorphisms in the complementary sequences of the microarray probes or mRNA splice variation and are not necessarily accompanied by transcription differences. True transcription variation however, can be caused by strong polymorphisms in promoter regions, premature stop mutations and even the complete absence of genes in one of the parental lines [39]. Both hybridization and true transcription variation will lead to strong *cis*-eQTLs which can subsequently be used as molecular markers, allowing the construction of high-resolution maps [40, 41].

### 2.2. Regulation *in trans*

The majority of differentially expressed genes will show a quantitative expression profile with complex inheritance patterns. This is because in general genes are regulated by many independent factors which can show up as *trans*-eQTLs. Because of the multiplicity of regulators and the often-observed epistasis between them, each *trans*-eQTL can have a relatively small effect. In addition, compared to the direct regulation of *cis*-eQTLs, the accumulation of stochastic variation in the expression of *trans*-regulated genes is indirectly also determined by the expression variation of one or more regulators. As a result the detected number of *trans-*eQTLs relative to the number of *cis*-eQTLs drops when the stringency for detection is increased [42].

Whereas *cis*-eQTLs are inherently associated with the gene in which they reside, a single gene can be responsible for the appearance of multiple *trans*-eQTLs throughout the genome. As a consequence the genome-wide distribution of *cis*-eQTLs is dependent on local gene density, although variation in chromatin structure can have an impact on the exposure of eQTLs. The distribution of *trans*-eQTLs however, can deviate substantially from what can be expected based on gene density. The identification of so-called hot spots, genomic regions with a high density of *trans*-eQTLs, can be explained by major regulators, *e.g.* transcription factors, which influence the expression of many downstream genes. In Arabidopsis this was illustrated by the large number of genes mapping to the *ERECTA* locus, a gene well-known for its pleiotropic effects on many morphological and developmental traits [22]. These findings suggest that the effects of key-regulators in gene expression are progressed to the phenotypic level. This was recently confirmed in a QTL study comparing transcript, protein and metabolite data with phenotypic traits [43]. Here, only a limited number of QTL hot spots with major, system-wide effects were detected, indicating that most of the genotypic variation is phenotypically buffered. These findings support the theory of biological robustness where hotspots indicate fragilities in this genetic buffering system [44]. Until now only a few reported hotspots have been verified and the number of detected hotspots is far from consistent between different genetical genomics studies. The latter reflects differences in the analyzed populations, species and conditions used and additionally might be the consequence of different statistical procedures used to identify eQTLs [45]. Because of the difficulties in cloning QTLs and the large biological relevance of hotspots, additional sources of information are often used to reduce the number of candidate genes or even predict the causal regulator. Such methods use information on gene ontology, (co-)expression, transcription factor binding sites and targets, ChIP-Seq and protein-protein interaction [46]. Together with computational methods such as regulatory modeling this can severely reduce the number of candidate genes and prioritize remaining candidates for further experimentation Fig. (**2**).

## 3. GENETICAL GENOMICS IN PLANTS

As discussed above many principles of genetic regulation are shared among different phylogenetic taxa. Not all species however are equally suited for large-scale experimentation. Sometimes evolutionary distances withhold translation of biological relevant findings in less conserved mechanisms, *e.g.* in yeast and Drosophila, or long generation times, inbreeding depression and moral and ethical issues hinder experimentation, *e.g.* in humans and other mammals. Plants, representing one of the largest kingdoms, are therefore often used to test concepts in genetic studies. The ease to generate large families from experimental crosses and the ability to store genotypes in the form of seeds or clonal propagation make plants ideal subjects to study the mechanistic basis of genetic regulation of traits.

### 3.1. Arabidopsis as a Reference Plant

The comprehensive resources which are available for *Arabidopsis thaliana*, such as a whole genome sequence, a large collection of natural variants and an ever-increasing number of molecular tools, made it the favorable model for genetical genomics research. As a non-obligate selfing species Arabidopsis combines the ability to cross-pollinate with high tolerance to inbreeding. Together with its short generation time and high reproductive success rate this enables the fast generation of large experimental populations such as Recombinant Inbred (RI) and Introgression Line (IL) populations. The availability and immortal character of such populations enable the accurate estimation of phenotypic values through replicated measurements and allows the testing of traits in different environments [47].

Traditionally QTL studies of ‘classical’ physiological traits in RIL populations are followed by mendelizing detected QTLs in near isogenic lines (NILs) for detailed analyses. By isolating QTLs from their genetic background it becomes much simpler to study their genetic effect and relate resulting phenotypes to other processes. Because it is expected that much of the phenotypic variation is the resultant of differences in gene expression and phenotypic perturbation in turn leads to transcriptional reprogramming, data mining for relationships between trait values and expression levels has become a common tool [48]. Very often mutants, knockouts or over-expression lines are used for these purposes, in which the effect of a single gene perturbation is tested on both the phenotypic and the expression level. For complex traits however, the causal genes leading to altered phenotypes are often not known and QTL analyses only identify genomic regions containing such genes. Nevertheless, using RIL populations to identify QTLs for a phenotypic trait and subsequently analyzing NILs for expression differences can be a powerful alternative to explore the functional relationship between genotype and phenotype (*e.g.* [49, 50]). Although the regions spanned by NILs can still contain hundreds of genes, of which many may display allelic variation between accessions, the *cis*-regulated genes are strong candidates explaining phenotypic diversity. High detection stringency can limit the number of differentially expressed genes to a reasonable number of candidate genes with strong local eQTLs [49].

The availability of a whole genome sequence in Arabidopsis provides unique opportunities, especially when multiple (epistatic) phenotypic QTLs are detected. Knowing the position of genes allows the identification of strong *cis*-regulated genes collocating with phenotypic QTLs. An early eQTL study in Arabidopsis analyzed genome-wide gene expression in a limited population of only 30 individuals, mimicking shoot regeneration conditions [21]. Two of the eQTL hotspots found coincided with shoot regeneration QTLs. The most significant eQTLs within these hotspot regions showed local chromosal linkage with their corresponding genes but the majority acted distantly. These results suggest that heritable *cis*-regulated expression changes of key-regulators determine *in trans* the expression of many genes related to differences in shoot regeneration efficiency between accessions. It also indicates that a long signaling cascade may exist between the causal genotypic polymorphism and the eventual phenotype.

In contrast to the former study it is not always necessary to combine phenotypic measurements with expression analysis. Often, many genes are known to play a role in the exposure of certain traits without knowledge about the genetic regulation of these genes. Specific analysis of such genes can help to identify common regulators. In the first genome-wide eQTL study in Arabidopsis, using a complete RIL population (162 lines), this concept was used to predict possible key-regulators of flowering time and circadian rhythms [22]. The benefits of using large populations for eQTL studies became also apparent in another study where expression analyses were performed in a RIL population of 211 individuals [24]. Whereas in the majority of cases only a single QTL could be detected per differentially expressed gene in the aforementioned studies, here the expression of many genes was controlled by multiple eQTLs. Moreover, a much larger fraction of genetically regulated genes was identified with a higher proportion of *trans*-regulated genes of which the vast majority exhibited small effects.

The studies performed in Arabidopsis show that the statistical power to detect eQTLs depends largely on population size. Nonetheless, it can not be excluded that differences in the analyzed tissues, developmental stages and populations used, such as parental variation, linkage distortion and recombination frequency, are responsible for part of the observed differences. All studies however, clearly demonstrated that variation in gene expression is for a large part genetically controlled, with much stronger effects of *cis*-eQTLs compared to *trans*-eQTLs. In general, *cis*-eQTLs also exhibit much higher heritability values and are obvious candidates to act as causal regulators of genes showing *trans*-eQTLs in the hotspots that could be detected in each of the discussed studies. The detection of regulatory loci for gene expression and the elucidation of their interaction networks might therefore provide the research community with a powerful tool to unravel the complex nature of natural variation in quantitative traits. 

### 3.2. Applications in Crop Species

Genetical genomics studies in Arabidopsis and other model species have shown the enormous benefits of the availability of an annotated genome sequence. However, until now full annotated genome sequence information for agronomical important species is only available for a limited number of species, including *Oryza sativa* [51, 52]*, Populus trichocarpa* [53],*Vitis vinifera* [54] and papaya [55]. This relatively low number of sequenced crop species can be explained by their often immense (polyploid) genome sizes and the highly repetitive nature of many crop genomes [56]. Nevertheless, sequence efforts for many more species, are ongoing and the increasing power of next-generation sequencing will soon lead to an almost unrestricted availability of genomic sequence information. Although an annotated genome is a valuable resource for the comparison of the genomic position of genes and their respective eQTLs, for most crop species this is not feasible yet. Nonetheless, several studies in crops for which genetic maps are available have shown that comprehensive genetical genomics approaches are possible without the need for annotated genome sequences [57-62].

Illustratively, one of the first large genetical genomics experiments was performed in an economically important species, *viz. Eucalyptus* [61]. QTL analysis of transcript levels of lignin-related genes showed that their mRNA abundance is regulated by two genetic loci coinciding with QTLs for stem diameter growth. Genetic mapping of some of the candidate genes showed that most of the lignin genes are under control of a *trans* eQTL hotspot which suggests that transcription of many of the genes in this pathway are under a higher level of coordinated control. A strong *cis*-regulated gene encoding S-adenosylmethionine synthase, collocating with the growth and transcription QTLs, was presented as the possible rate limiting step in lignin biosynthesis and as such a strong candidate for the observed QTLs [61].

In some crops the required availability of genomic sequence data for large-scale classification of *cis/trans* eQTLs can be circumvented by making use of synteny with other species. In wheat, synteny with rice was used to assist the physical mapping of wheat genes [63]. A genetical genomics approach was conducted in a segregating population of 41 doubled haploid (DH) lines to study agronomic important seed quality parameters. Assuming that the most significantly different expressed genes were *cis*-regulated, a selection of genes was subjected to synteny analyses. This enabled the positioning of genes with biological relevant linkage to phenotypic traits in a species for which full genome sequence is not available yet.

In the absence of genome-wide micro-arrays, expressed sequence tag (EST) libraries allow the construction of species specific sub genome-scale microarrays. In maize, cell-wall digestibility, which is the major target for improving the feeding value of forage maize, was analyzed in a RIL population [62]. In addition forty extreme RIL lines were hybridized on a small microarray with 439 preselected candidate ESTs for cell-wall digestibility genes for which 89 eQTLs could be mapped. One eQTL hotspot collocated with a cell-wall digestibility related QTL [62]. The application of genetical genomics approaches can be of special interest here when the detection of eQTLs is combined with ASE assays. The thus identified *cis*-regulated genes can then be positioned on the genetic map where they may serve as candidate genes underpinning phenotypic QTLs.

An interesting alternative for species for which no (EST) sequence information is available at all, and hence no microarrays can be produced, is a gel-based cDNA-AFLP approach [64]. Here AFLP band intensities, reflecting expression differences, are profiled for a large proportion of the transcribed gene pool enabling standard eQTL analyses procedures. AFLP bands showing significant eQTLs can subsequently be sequenced to obtain the identity of the gene from which the fragment derived. Additionally, the cDNA-AFLPs can be used to construct a genetic map. 

The examples given above show that genetical genomics is not necessarily restricted to model species but can be applied to any species in which experimental crosses are possible even in the absence of genomic sequence or genetic map information. The potential of combining phenotypic QTL analysis with gene expression traits is shown in a number of economically important species, e.g. *Populus* [57], cotton [58], rice [59] and sunflower [60]. The application of genetical genomics is particulary promising in breeding programs of crops that take advantage of hybrid vigour. The eQTLs involved in heterosis will segregate consistently in a F_1_ backcross population thereby identifying valuable targets for marker assisted breeding for the best combination of alleles in the parents of the hybrid [65].

## 4. NETWORK RECONSTRUCTION

Genetical genomics harbors the potential to dissect the genetic regulation of a specific biological process. Therefore, methods to reconstruct regulatory networks from eQTL data have obtained much attention. Prioritizing on *cis-*eQTLs that collocate with a phenotypic QTL is a valuable approach for causal gene discovery, but in many cases little is known about the global regulation, interaction and function of genes that control a biological process. Identification of a set of genes with a *trans-*eQTL at an identical position can help to dissect genetic variation that is influencing an entire pathway and can lead to the identification of initiating polymorphisms upstream in a network [66]. Questions about the regulatory level at which *trans* polymorphisms act in the global gene expression network and what their effect is on phenotypic variation and heritability can only be addressed when eQTLs are further dissected.

With a genetical genomics approach one can use the natural genetic variation as a source of perturbations to elucidate the structure of networks. In a summation approach eQTLs for all genes in the analysis are simply superimposed to identify common regions which control many genes [14]. Such an approach does not require any *a priori* network information but applies subsequent Gene Set Enrichment Analysis (GSEA) using gene onthology (GO) annotation or other descriptors to test whether selected genes share a common biological function [67]. If the network under study is largely known or at least predicted, an *a priori* analysis can be performed. Here, the expression levels of individual genes in the network are converted into a common measure for the expression level of the entire network which is then used as the trait for QTL analysis. This strategy was tested in an Arabidopsis RIL population for 20 gene expression networks and resulted in statistically significant network variation for eighteen of the 20 predefined networks [68]. Combining summation, GSEA and *a priori* network analyses allows the generation of a more specific hypothesis about phenotypic effects of network eQTLs. In a study using 175 genes, selected to be involved in regulation of flowering and circadian rhythms, 83 genes showed an eQTL [22]. By combining co-expression analysis, which becomes feasible for microarray compendia of large populations, and positional information of genes and their eQTLs, it was possible to construct regulatory networks of key-regulators and their target genes, predicting unknown relationships and confirming common knowledge.

Pre-selection of known pathways can obviously hinder the elucidation of novel networks in a species, for which much effort is made to develop methods to translate eQTL data into network information using an *a posteriori* approach. As the precise balance of active components within a tightly controlled biological pathway is in part maintained by coordinately regulated gene expression, this creates possibilities to model networks by exploring co-expression of untargeted genes. To validate this hypothesis, gene expression in liver from a population of 60 mice with variation in diabetes susceptibility was analyzed [69]. The combination of correlation analysis across a genetic dimension and linkage mapping enabled the identification of regulatory networks, functional predictions for uncharacterized genes and characterization of novel members of known pathways. A similar approach in *Drosophila*, complemented with information about gene ontology, tissue specific expression and transcription factor binding sites, led to the construction of multiple interconnected networks with biological relevance for phenotypic traits [70].

Understanding the mechanisms underlying trait regulation requires the identification of specific causal polymorphisms. For this purpose sophisticated self-learning algorithms have been developed which make use of conservation, type and position of a particular SNP to prioritize causal regulators by estimating the likeliness that it plays a causal role in gene expression variation [71]. Extending such approaches might also provide the means to distinguish whether variation in gene expression or a regulatory network is the cause or a consequence of an altered phenotype, resulting in the construction of probabilistic directional networks [72]. Defining such causal networks is also known as reverse engineering, because it aims at understanding how the system works as an integrated whole instead of only defining the functionally related components.

## 5. NEXT LEVEL NETWORKS: INTEGRATION OF OTHER ‘OMICS’ DATA

Although phenotypic variation can be partly explained by genetic variation in gene expression, this alone does not fully cover the possible differences in the regulatory mechanisms of an organism. Similar transcript levels of allelic gene variants can still result in varying protein levels because of variance in translational activity, protein degradation and post-translational modifications [73]. Furthermore, variation in coding sequences can alter protein function resulting in a flexible metabolome in terms of chemical structure and function [74]. Integrating ‘omics’ data such as gene expression, SNPs, metabolomics and proteomics in genetic studies can therefore reduce the number of candidate genes for a given QTL from hundreds to a manageable list without excluding regulatory mechanisms *a priori*. Because of the analytical complexity in analyzing large numbers of protein samples, genetical proteomics studies are limited (*e.g.* [75]) but advances made in biochemical detection have already enabled the large-scale untargeted genetic analysis of metabolic content [76-78].

The complex relationship between different levels of regulation was illustrated in a study integrating parallel QTL analyses of the expression of genes, activity of encoded enzymes and metabolites involved in primary carbohydrate metabolism [79]. It could be shown that regulation acted on each of the intermediary levels of the path from genotype to phenotype. Although seemingly specific independent regulation could be observed for each analyzed trait, a strong interconnectivity existed between them resulting in coherent systematic differences between population individuals.

The importance of the tight regulation of such an essential component in plant development as primary metabolism was also demonstrated in an Arabidopsis RIL population where plant biomass was related to the metabolic profile [80]. Again, no relationship could be observed between individual metabolites and plant growth but a strong canonical correlation was observed between biomass and a specific combination of metabolites in central metabolism. The power of large-scale metabolomic profiling combined with detailed morphological analysis was also shown in tomato [77]. Significant QTLs could be detected for the accumulation of a large number of primary metabolites together with loci that modify yield-associated traits. With this information a correlation network revealing associations between phenotype, metabolic content and nutritional value could be generated. These studies show that analyzing phenotypic traits and metabolic profiles in a genetic mapping population has great potential for the generation of biomarkers in breeding programs.

Whereas primary metabolites are essential in central metabolism governing growth and development, plants also accumulate large amounts of secondary metabolites. These are believed to be less essential but may play an important role in the adaptation of plants to local environments. Since Arabidopsis can be found in a wide variety of habitats, variation in secondary metabolism might explain much of the evolutionary success of the species. A large untargeted screen of variation in secondary metabolic composition indeed revealed a high proportion of genetically controlled compounds [76]. The highly flexible nature of the metabolome was clearly shown by the fact that more than one-third of the compounds present in the RILs were not detected in either parent but were the result of recombination in biosynthesis pathways. The genetic information obtained from such studies is of great value for the construction of molecular biosynthesis networks, especially if they can be combined with expression data.

This strategy was applied in the genetic analysis of glucosinolate biosynthetic networks which were studied at both the transcriptional and metabolic level [81]. In all cases, variation in gene expression also affected the accumulation of metabolites but epistasis was detected more frequently for metabolic traits as compared to transcript traits. Within such an *a priori* defined framework it was possible to identify and unravel complex regulatory mechanisms like metabolic feedback loops in which metabolic content regulated gene expression and vice versa. 

The examples discussed here highlight the technological advances made in high-throughput characterization of the transcriptome, the proteome and metabolome which enables an integrated multidisciplinary approach to unravel the regulatory mechanisms involved in natural variation of complex traits. 

## 6. FUTURE CHALLENGES

Although much progress is being made in understanding the influence of genetic factors on a biological system we still have limited understanding of the interplay between environment and genetic factors. The discovery of molecular networks with genetical genomics approaches is often limited to a single experimental condition. An interesting concept, called generalized genetical genomics, studies controlled environmental perturbations combined with genetical genomics [82]. This generalization of genetical genomics will detect how the response to environmental changes is influenced by the genotype (*i.e.* genotype x environment interactions). Here, spatial and temporal variation can also be regarded as different environments since specific tissues and developmental stages often determine the biological context in which regulatory networks function.

The advances in next generation sequence technology will continue to produce huge amounts of sequence data. Good examples are the human 1000 [83] and the 1001 Arabidopsis [84] genome projects which aim at resequencing over 1000 different humans and accessions respectively. However, *de novo* sequencing of economically important or phylogenetic strategically chosen species is of equal importance. The accumulation of genomic information, in combination with genetical genomics approaches, will enable the precise definition of functional important polymorphisms and their role in adaptation to changing environments and species formation. Having access to complete genome sequences also enables the generation of full genome tiling arrays for different (crop) species, which have been proven to be very useful for expression profiling [85, 86]. When used within a genetical genomics approach this offers unique features to elucidate the genetics behind the mechanistic basis of transcriptional differences. For Arabidopsis for instance, a SNPtile microarray was developed harboring tiling probes covering both strands of the genome and in addition probes for genome-wide detection of SNPs and CpG methylation [38]. A properly designed genetical genomics study using such arrays might reveal genetic variation for gene expression, alternative splicing, regulation of *cis*-natural antisense transcripts, allele specific expression and epigenetic regulation. 

As a result of developments in SNP-discovery and platforms for genotyping large collections of individuals, the application of Linkage disequilibrium (LD) mapping for complex traits has become within reach. LD or association mapping detects the non-random inheritance of alleles at separate loci located on the same chromosome. In an experimental F_2_ or RIL population the genetic variation is limited to the extent of natural variation present in the parental lines and resolution depends on the recombination frequency within and size of the population. In contrast LD mapping makes use of large collections of natural (wild) accessions or elite breeding lines, sampling a much larger fraction of the natural variation present within a species. Moreover, it benefits from the much higher frequency of recombination events accumulated during the evolutionary history of a species allowing higher resolution mapping [87]. The extent of LD varies between species and traits analyzed but the gain in resolution relative to experimental populations lies in the order of magnitudes, equally increasing the need for dense marker spacing to enable genome wide scans [88]. This high number of necessary markers has always been a big limitation for LD mapping but next generation sequencing will tremendously increase the available number of markers. Therefore, we see great potential for phenotyping and expression profiling of LD populations to detect causal genes for natural variation and enable marker-assisted selection in breeding programs.

## 7. CONCLUDING REMARKS

Since its introduction the concept of genetical genomics has proven to be a powerful approach to dissect genetic variation. Studies in crop species revealed major *cis*-eQTLs which collocated with important phenotypic traits and therefore will facilitate faster crop improvement. The genetical genomics studies in model species help to understand the extent of genetic variation and much effort is spent to develop statistical tools for building and elucidating causal networks. Recent developments of inexpensive high-throughput sequencing techniques and next generation tiling microarrays will soon create opportunities to extend genetical genomics to unravel the genetic variation of gene expression, alternative splicing, allele specific expression and epigenetic polymorphisms. Similarly, continuing technological developments have increased the power of both proteomic and metabolomic approaches. Integration of phenotypic, genetic, transcriptomic, proteomic and metabolomic data will enable accurate and detailed network reconstruction. This will ultimately result in the elucidation of the molecular pathways involved in complex phenotypic traits.

## Figures and Tables

**Fig. (1):**
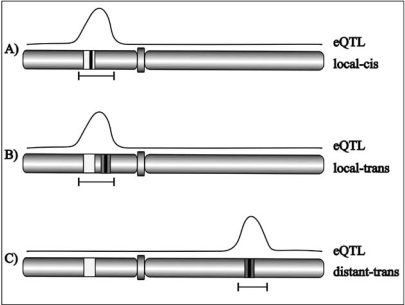
Classification of eQTLs (solid line) based on the expression of the gene under study (light grey box) and location of the causal polymorphism (black bar). **A**) local *cis*-eQTL, result from allelic variation of the gene under study. **B**) local *trans*-eQTL, causal polymorphism within the eQTL confidence interval but not inside the gene under study (no allele specific expression). **C**) distant *trans*-eQTL, gene under study is located outside the confidence interval of its eQTL.

**Fig. (2):**
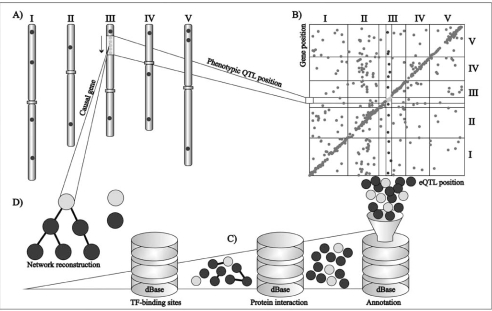
Schematic representation of candidate gene selection with a genetical genomics approach. **A**) Five chromosomes of Arabidopsis thaliana (I-V) with a QTL support interval for a phenotypic trait indicated on chromosome III. **B**) eQTL plot showing the position of genes and their corresponding eQTLs. Genes within the support interval can be causal for the observed QTL, of which *cis*-regulated genes, indicated in light grey, represent the strongest candidates. Genes outside the QTL support interval but regulated *in trans* by the same locus, indicated in dark grey, might be involved in the biological process under study and represent downstream effects of the QTL. **C**) Available prior information of the selected genes such as gene onthology and biological interaction data can assist in limiting the number of genes to those most likely involved in the trait under study. **D**) Connectivity between the remaining genes is than used to construct maximum likelihood hypothetical regulatory networks which will suggest the strongest candidate regulator gene causal for the observed phenotypic QTL.

## References

[1] Galbraith D.W (2006). DNA microarray analyses in higher plants. OMICS.

[2] Joosen R.V.L, Cordewener J, Supena E.D.J, Vorst O, Lammers M, Maliepaard C, Zeilmaker T, Miki B, America T, Custers J, Boutilier K (2007). Combined transcriptome and proteome analysis identifies pathways and markers associated with the establishment of rapeseed microspore-derived embryo development. Plant Physiol.

[3] Brazma A, Parkinson H, Sarkans U, Shojatalab M, Vilo J, Abeygunawardena N, Holloway E, Kapushesky M, Kemmeren P, Lara G.G, Oezcimen A, Rocca-Serra P, Sansone S.A (2003). ArrayExpress - A public repository for microarray gene expression data at the EBI. Nucleic Acids Res.

[4] Toufighi K, Brady S. M, Austin R, Ly E, Provart N .J (2005). The botany array resource: e-Northerns, expression angling, and promoter analyses. Plant J.

[5] Zimmermann P, Hennig L, Gruissem W (2005). Gene-expression analysis and network discovery using Genevestigator. Trends Plant Sci.

[6] Fujita A, Sato J, Garay-Malpartida H. M, Sogayar M. C, Farreira C. E, Miyano S (2008). Modeling nonlinear gene regulatory networks from time series gene expression data. J. Bioinform. Comput. Biol.

[7] Gupta A, Maranas C. D, Albert R (2006). Elucidation of directionality for co-expressed genes: Predicting intra-operon termination sites. Bioinformatics.

[8] Alonso-Blanco C, Koornneef M (2000). Naturally occurring variation in Arabidopsis: an underexploited resource for plant genetics. Trends Plant Sci.

[9] Ashikari M, Matsuoka M (2006). Identification, isolation and pyramiding of quantitative trait loci for rice breeding. Trends Plant Sci.

[10] Salvi S, Tuberosa R (2005). To clone or not to clone plant QTLs: Present and future challenges. Trends Plant Sci.

[11] Jansen R.C, Nap J.P (2001). Genetical genomics: the added value from segregation. Trends Genet.

[12] Keurentjes J.J.B, Koornneef M, Vreugdenhil D (2008). Quantitative genetics in the age of omics. Curr. Opin. Plant Biol.

[13] Brem R.B, Yvert G, Clinton R, Kruglyak L (2002). Genetic dissection of transcriptional regulation in budding yeast. Science.

[14] Schadt E. E, Monks S. A, Drake T. A, Lusis A. J, Che N, Colinayo V, Ruff T. G, Milligan S. B, Lamb J. R, Cavet G, Linsley P. S, Mao M, Stoughton R. B, Friend S. H (2003). Genetics of gene expression surveyed in maize, mouse and man. Nature.

[15] Yvert G, Brem R.B, Whittle J, Akey J.M, Foss E, Smith E.N, Mackelprang R, Kruglyak L (2003). Trans-acting regulatory variation in Saccharomyces cerevisiae and the role of transcription factors. Nat. Genet.

[16] Leach L.J, Zhang Z, Lu C, Kearsey M.J, Luo Z (2007). The role of cis-regulatory motifs and genetical control of expression in the divergence of yeast duplicate genes. Mol. Biol. Evol.

[17] Bing N, Hoeschele I (2005). Genetical genomics analysis of a yeast segregant population for transcription network inference. Genetics.

[18] Li Y, Alvarez O.A, Gutteling E.W, Tijsterman M, Fu J, Riksen J. A, Hazendonk E, Prins P, Plasterk R.H, Jansen R.C, Breitling R, Kammenga J.E (2006). Mapping determinants of gene expression plasticity by genetical genomics in C. elegans. PLoS Genet.

[19] Wittkopp P.J, Haerum B.K, Clark A.G (2004). Evolutionary changes in cis and trans gene regulation. Nature.

[20] Hsieh W.P, Passador-Gurgel G, Stone E.A, Gibson G (2007). Mixture modeling of transcript abundance classes in natural populations. Genome Biol.

[21] DeCook R, Lall S, Nettleton D, Howell S. H (2006). Genetic regulation of gene expression during shoot development in Arabidopsis. Genetics.

[22] Keurentjes J.J.B, Fu J, Terpstra I.R, Garcia J.M, van den Ackerveken G, Snoek L.B, Peeters A.J, Vreugdenhil D, Koornneef M, Jansen R.C (2007). Regulatory network construction in Arabidopsis by using genome-wide gene expression quantitative trait loci. Proc. Natl. Acad. Sci. USA.

[23] Potokina E, Druka A, Luo Z, Wise R, Waugh R, Kearsey M (2008). Gene expression quantitative trait locus analysis of 16 000 barley genes reveals a complex pattern of genome-wide transcriptional regulation. Plant J.

[24] West M.A.L, Kim K, Kliebenstein D.J, Van Leeuwen H, Michelmore R.W, Doerge R.W, St. Clair D.A (2007). Global eQTL mapping reveals the complex genetic architecture of transcript-level variation in Arabidopsis. Genetics.

[25] Bystrykh L, Weersing E, Dontje B, Sutton S, Pletcher M.T, Wiltshire T, Su A.I, Vellenga E, Wang J, Manly K.F, Lu L, Chesler E.J, Alberts R, Jansen R.C, Williams R.W, Cooke M.P, De Haan G (2005). Uncovering regulatory pathways that affect hematopoietic stem cell function using 'genetical genomics'. Nat. Genet.

[26] Chesler E. J, Lu L, Shou S, Qu Y, Gu J, Wang J, Hsu H.C, Mountz J.D, Baldwin N.E, Langston M.A, Threadgill D.W, Manly K.F, Williams R.W (2005). Complex trait analysis of gene expression uncovers polygenic and pleiotropic networks that modulate nervous system function. Nat. Genet.

[27] Hubner N, Wallace C.A, Zimdahl H, Petretto E, Schulz H, Maciver F, Mueller M, Hummel O, Monti J, Zidek V, Musilova A, Kren V, Causton H, Game L, Born G, Schmidt S, Müller A, Cook S.A, Kurtz T.W, Whittaker J, Pravenec M, Aitman T. J (2005). Integrated transcriptional profiling and linkage analysis for identification of genes underlying disease. Nat. Genet.

[28] Monks S.A, Leonardson A, Zhu H, Cundiff P, Pietrusiak P, Edwards S, Phillips J.W, Sachs A, Schadt E.E (2004). Genetic inheritance of gene expression in human cell lines. Am. J. Hum. Genet.

[29] Stranger B.E, Nica A.C, Forrest M.S, Dimas A, Bird C.P, Beazley C, Ingle C.E, Dunning M, Flicek P, Koller D, Montgomery S, Tavaré S, Deloukas P, Dermitzakis E. T (2007). Population genomics of human gene expression. Nat. Genet.

[30] Dixon A.L, Liang L, Moffatt M.F, Chen W, Heath S, Wong K.C.C, Taylor J, Burnett E, Gut I, Farrall M, Lathrop G.M, Abecasis G.R, Cookson W.O.C (2007). A genome-wide association study of global gene expression. Nat. Genet.

[31] Göring H.H.H, Curran J.E, Johnson M.P, Dyer T.D, Charlesworth J, Cole S.A, Jowett J.B.M, Abraham L.J, Rainwater D.L, Comuzzie A.G, Mahaney M.C, Almasy L, MacCluer J.W, Kissebah A.H, Collier G.R, Moses E.K, Blangero J (2007). Discovery of expression QTLs using large-scale transcriptional profiling in human lymphocytes. Nat. Genet.

[32] Myers A.J, Gibbs J. R, Webster J. A, Rohrer K, Zhao A, Marlowe L, Kaleem M, Leung D, Bryden L, Nath P, Zismann V. L, Joshipura K, Huentelman M. J, Hu-Lince D, Coon K. D, Craig D. W, Pearson J. V, Holmans P, Heward C. B, Reiman E. M, Stephan D, Hardy J (2007). A survey of genetic human cortical gene expression. Nat. Genet.

[33] Emilsson V, Thorleifsson G, Zhang B, Leonardson A. S, Zink F, Zhu J, Carlson S, Helgason A, Walters G. B, Gunnarsdottir S, Mouy M, Steinthorsdottir V, Eiriksdottir G. H, Bjornsdottir G, Reynisdottir I, Gudbjartsson D, Helgadottir A, Jonasdottir A, Styrkarsdottir U, Gretarsdottir S, Magnusson K. P, Stefansson H, Fossdal R, Kristjansson K, Gislason H. G, Stefansson T, Leifsson B. G, Thorsteinsdottir U, Lamb J. R, Gulcher J. R, Reitman M. L, Kong A, Schadt E. E, Stefansson K (2008). Genetics of gene expression and its effect on disease. Nature.

[34] Mitchell-Olds T, Schmitt J (2006). Genetic mechanisms and evolutionary significance of natural variation in Arabidopsis. Nature.

[35] Rockman M.V, Kruglyak L (2006). Genetics of global gene expression. Nat. Rev. Genet.

[36] Gibson G, Weir B (2005). The quantitative genetics of transcription. Trends Genet.

[37] Cowles C.R, Hirschhorn J.N, Altshuler D, Lander E.S (2002). Detection of regulatory variation in mouse genes. Nat. Genet.

[38] Zhang X, Richards E.J, Borevitz J.O (2007). Genetic and epigenetic dissection of cis regulatory variation. Curr. Opin. Plant Biol.

[39] Gilad Y, Borevitz J (2006). Using DNA microarrays to study natural variation. Curr. Opin. Genet. Dev.

[40] Borevitz J.O, Chory J (2004). Genomics tools for QTL analysis and gene discovery. Curr. Opin. Plant Biol.

[41] West M.A.L, Van Leeuwen H, Kozik A, Kliebenstein D.J, Doerge R.W, St. Clair D.A, Michelmore R.W (2006). High-density haplotyping with microarray-based expression and single feature polymorphism markers in Arabidopsis. Genome Res.

[42] Doss S, Schadt E.E, Drake T.A, Lusis A.J (2005). Cis-acting expression quantitative trait loci in mice. Genome Res.

[43] Fu J, Keurentjes J.J.B, Bouwmeester H, America T, Verstappen F.W, Ward J.L, Beale M.H, de Vos R.C, Dijkstra M, Scheltema R.A, Johannes F, Koornneef M, Vreugdenhil D, Breitling R, Jansen R.C (2009). System-wide molecular evidence for phenotypic buffering in Arabidopsis. Nat. Genet.

[44] Kitano H (2004). Biological robustness. Nat. Rev. Genet.

[45] Breitling R, Li Y, Tesson B.M, Fu J, Wu C, Wiltshire T, Gerrits A, Bystrykh L.V, De Haan G, Su A.I, Jansen R.C (2008). Genetical genomics: Spotlight on QTL hotspots. PLoS Genet.

[46] Zhu J, Zhang B, Smith EN, Drees B, Brem R.B, Kruglyak L, Bumgarner R. E, Schadt E.E (2008). Integrating large-scale functional genomic data to dissect the complexity of yeast regulatory networks. Nat. Genet.

[47] Paran I, Zamir D (2003). Quantitative traits in plants: Beyond the QTL. Trends Genet.

[48] Weckwerth W, Loureiro M.E, Wenzel K, Fiehn O (2004). Differential metabolic networks unravel the effects of silent plant phenotypes. Proc. Natl. Acad. Sci. USA.

[49] Juenger T. E, Wayne T, Boles S, Symonds V. V, McKay J, Coughlan S.J (2006). Natural genetic variation in whole-genome expression in Arabidopsis thaliana: The impact of physiological QTL introgression. Mol. Ecol.

[50] Juenger T.E, McKay J.K, Hausmann N, Keurentjes J.J.B, Sen S, Stowe K.A, Dawson T.E, Simms EL, Richards J.H (2005). Identification and characterization of QTL underlying wholeplant physiology in Arabidopsis thaliana: ?13C, stomatal conductance and transpiration efficiency. Plant Cell Environ.

[51] Goff S.A, Ricke D, Lan T. H, Presting G, Wang R, Dunn M, Glazebrook J, Sessions A, Oeller P, Varma H, Hadley D, Hutchison D, Martin C, Katagiri F, Lange B. M, Moughamer T, Xia Y, Budworth P, Zhong J, Miguel T, Paszkowski U, Zhang S, Colbert M, Sun W. L, Chen L, Cooper B, Park S, Wood T. C, Mao L, Quail P, Wing R, Dean R, Yu Y, Zharkikh A, Shen R, Sahasrabudhe S, Thomas A, Cannings R, Gutin A, Pruss D, Reid J, Tavtigian S, Mitchell J, Eldredge G, Scholl T, Miller R. M, Bhatnagar S, Adey N, Rubano T, Tusneem N, Robinson R, Feldhaus J, Macalma T, Oliphant A, Briggs S (2002). A draft sequence of the rice genome (Oryza sativa L. ssp. japonica). Science.

[52] Yu J, Hu S, Wang J, Wong G. K. S, Li S, Liu B, Deng Y, Dai L, Zhou Y, Zhang X, Cao M, Liu J, Sun J, Tang J, Chen Y, Huang X, Lin W, Ye C, Tong W, Cong L, Geng J, Han Y, Li L, Li W, Hu G, Li J, Liu Z, Qi Q, Li T, Wang X, Lu H, Wu T, Zhu M, Ni P, Han H, Dong W, Ren X, Feng X, Cui P, Li X, Wang H, Xu X, Zhai W, Xu Z, Zhang J, He S, Xu J, Zhang K, Zheng X, Dong J, Zeng W, Tao L, Ye J, Tan J, Chen X, He J, Liu D, Tian W, Tian C, Xia H, Bao Q, Li G, Gao H, Cao T, Zhao W, Li P, Chen W, Zhang Y, Hu J, Liu S, Yang J, Zhang G, Xiong Y, Li Z, Mao L, Zhou C, Zhu Z, Chen R, Hao B, Zheng W, Chen S, Guo W, Tao M, Zhu L, Yang H (2002). A draft sequence of the rice genome (Oryza sativa L. ssp. indica). Science.

[53] Tuskan G. A, Difazio S, Jansson S, Bohlmann J, Grigoriev I, Hellsten U, Putnam N, Ralph S, Rombauts S, Salamov A, Schein J, Sterck L, Aerts A, Bhalerao R. R, Bhalerao R. P, Blaudez D, Boerjan W, Brun A, Brunner A, Busov V, Campbell M, Carlson J, Chalot M, Chapman J, Chen G. L, Cooper D, Coutinho P. M, Couturier J, Covert S, Cronk Q, Cunningham R, Davis J, Degroeve S, Dejardin A, Depamphilis C, Detter J, Dirks B, Dubchak I, Duplessis S, Ehlting J, Ellis B, Gendler K, Goodstein D, Gribskov M, Grimwood J, Groover A, Gunter L, Hamberger B, Heinze B, Helariutta Y, Henrissat B, Holligan D, Holt R, Huang W, Islam-Faridi N, Jones S, Jones-Rhoades M, Jorgensen R, Joshi C, Kangasjarvi J, Karlsson J, Kelleher C, Kirkpatrick R, Kirst M, Kohler A, Kalluri U, Larimer F, Leebens-Mack J, Leple J C, Locascio P, Lou Y, Lucas S, Martin F, Montanini B, Napoli C, Nelson D R, Nelson C, Nieminen K, Nilsson O, Pereda V, Peter G, Philippe R, Pilate G, Poliakov A, Razumovskaya J, Richardson P, Rinaldi C, Ritland K, Rouze P, Ryaboy D, Schmutz J, Schrader J, Segerman B, Shin H, Siddiqui A, Sterky F, Terry A, Tsai C J, Uberbacher E, Unneberg P, Vahala J, Wall K, Wessler S, Yang G, Yin T, Douglas C, Marra M, Sandberg G, Van de Peer Y, Rokhsar D (2006). The genome of black cottonwood, Populus trichocarpa (Torr. & Gray). Science.

[54] Jaillon O, Aury J. M, Noel B, Policriti A, Clepet C, Casagrande A, Choisne N, Aubourg S, Vitulo N, Jubin C, Vezzi A, Legeai F, Hugueney P, Dasilva C, Horner D, Mica E, Jublot D, Poulain J, Bruyere C, Billault A, Segurens B, Gouyvenoux M, Ugarte E, Cattonaro F, Anthouard V, Vico V, Del Fabbro C, Alaux M, Di Gaspero G, Dumas V, Felice N, Paillard S, Juman I, Moroldo M, Scalabrin S, Canaguier A, Le Clainche I, Malacrida G, Durand E, Pesole G, Laucou V, Chatelet P, Merdinoglu D, Delledonne M, Pezzotti M, Lecharny A, Scarpelli C, Artiguenave F, Pe M E, Valle G, Morgante M, Caboche M, Adam-Blondon A F, Weissenbach J, Quetier F, Wincker P (2007). The grapevine genome sequence suggests ancestral hexaploidization in major angiosperm phyla. Nature.

[55] Ming R, Hou S, Feng Y, Yu Q, Dionne-Laporte A, Saw J. H, Senin P, Wang W, Ly B. V, Lewis K. L. T, Salzberg S. L, Feng L, Jones M. R, Skelton R. L, Murray J. E, Chen C, Qian W, Shen J, Du P, Eustice M, Tong E, Tang H, Lyons E, Paull R. E, Michael T. P, Wall K, Rice D. W, Albert H, Wang M. L, Zhu Y. J, Schatz M, Nagarajan N, Acob R. A, Guan P, Blas A, Wai C. M, Ackerman C. M, Ren Y, Liu C, Wang J, Na J. K, Shakirov E. V, Haas B, Thimmapuram J, Nelson D, Wang X, Bowers J. E, Gschwend A. R, Delcher A. L, Singh R, Suzuki J. Y, Tripathi S, Neupane K, Wei H, Irikura B, Paidi M, Jiang N, Zhang W, Presting G, Windsor A, Navajas-Pérez R, Torres M. J, Feltus F. A, Porter B, Li Y, Burroughs A. M, Luo M. C, Liu L, Christopher D. A, Mount S. M, Moore P. H, Sugimura T, Jiang J, Schuler M. A, Friedman V, Mitchell-Olds T, Shippen D. E, Depamphilis C. W, Palmer J. D, Freeling M, Paterson A. H, Gonsalves D, Wang L, Alam M (2008). The draft genome of the transgenic tropical fruit tree papaya (Carica papaya Linnaeus). Nature.

[56] Burke J. M, Burger J. C, Chapman M. A (2007). Crop evolution: from genetics to genomics. Curr. Opin. Genet. Dev.

[57] Street N. R, Skogstrom O, Sjodin A, Tucker J, Rodriguez-Acosta M, Nilsson P, Jansson S, Taylor G (2006). The genetics and genomics of the drought response in Populus. Plant J.

[58] An C, Saha S, Jenkins J. N, Scheffler B. E, Wilkins T. A, Stelly D. M (2007). Transcriptome profiling, sequence characterization, and SNP-based chromosomal assignment of the EXPANSIN genes in cotton. Mol. Genet. Genomics.

[59] Venu R. C, Jia Y, Gowda M, Jia M. H, Jantasuriyarat C, Stahlberg E, Li H, Rhineheart A, Boddhireddy P, Singh P, Rutger N, Kudrna D, Wing R, Nelson J. C, Wang G. L (2007). RL-SAGE and microarray analysis of the rice transcriptome after Rhizoctonia solani infection. Mol. Genet. Genomics.

[60] Poormohammad Kiani S, Grieu P, Maury P, Hewezi T, Gentzbittel L, Sarrafi A (2007). Genetic variability for physiological traits under drought conditions and differential expression of water stress-associated genes in sunflower (Helianthus annuus L.). Theor. Appl. Genet.

[61] Kirst M, Myburg A. A, De Leon J. P, Kirst M. E, Scott J, Sederoff R (2004). Coordinated genetic regulation of growth and lignin revealed by quantitative trait locus analysis of cDNA microarray data in an interspecific backcross of eucalyptus. Plant Physiol.

[62] Shi C, Uzarowska A, Ouzunova M, Landbeck M, Wenzel G, Lübberstedt T (2007). Identification of candidate genes associated with cell wall digestibility and eQTL (expression quantitative trait loci) analysis in a Flint x Flint maize recombinant inbred line population. BMC Genomics.

[63] Jordan M. C, Somers D. J, Banks T. W (2007). Identifying regions of the wheat genome controlling seed development by mapping expression quantitative trait loci. Plant Biotechnol. J.

[64] Vuylsteke M, Van Den Daele H, Vercauteren A, Zabeau M, Kuiper M (2006). Genetic dissection of transcriptional regulation by cDNA-AFLP. Plant J.

[65] Kirst M, Basten C.J, Myburg A.A, Zeng Z.B, Sederoff R.R (2005). Genetic architecture of transcript-level variation in differentiating xylem of a eucalyptus hybrid. Genetics.

[66] Hansen B.G, Halkier B.A, Kliebenstein DJ (2008). Identifying the molecular basis of QTLs: eQTLs add a new dimension. Trends Plant Sci.

[67] Subramanian A, Tamayo P, Mootha V. K, Mukherjee S, Ebert B. L, Gillette M. A, Paulovich A, Pomeroy S. L, Golub T. R, Lander E. S, Mesirov J. P (2005). Gene set enrichment analysis: a knowledge-based approach for interpreting genome-wide expression profiles. Proc. Natl. Acad. Sci. USA.

[68] Kliebenstein D. J, West M. A. L, van Leeuwen H, Loudet O, Doerge R. W, St. Clair D. A (2006). Identification of QTLs controlling gene expression networks defined a priori. BMC Bioinformatics.

[69] Lan H, Chen M, Flowers J. B, Yandell B. S, Stapleton D. S, Mata C. M, Mui E. T. K, Flowers M. T, Schueler K. L, Manly K. F, Williams R. W, Kendziorski C, Attie A. D (2006). Combined expression trait correlations and expression quantitative trait locus mapping. PLoS Genet.

[70] Ayroles J.F, Carbone M.A, Stone E.A, Jordan K.W, Lyman R.F, Magwire M.M, Rollmann S.M, Duncan L.H, Lawrence F, Anholt R.R.H, Mackay T. F.C (2009). Systems genetics of complex traits in Drosophila melanogaster. Nat. Genet.

[71] Lee S. I, Dudley A.M, Drubin D, Silver P.A, Krogan N.J, Pe'er D, Koller D (2009). Learning a prior on regulatory potential from eQTL data. PLoS Genet.

[72] Rockman M.V (2008). Reverse engineering the genotype-phenotype map with natural genetic variation. Nature.

[73] Stylianou I. M, Affourtit J. P, Shockley K. R, Wilpan R. Y, Abdi F. A, Bhardwaj S, Rollins J, Churchill G. A, Paigen B (2008). Applying gene expression, proteomics and single-nucleotide polymorphism analysis for complex trait gene identification. Genetics.

[74] Keurentjes J.J.B (2009). Genetical metabolomics: closing in on phenotypes. Curr. Opin. Plant Biol.

[75] Chevalier F, Martin O, Rofidal V, Devauchelle A. D, Barteau S, Sommerer N, Rossignol M (2004). Proteomic investigation of natural variation between Arabidopsis ecotypes. Proteomics.

[76] Keurentjes J. J. B, Fu J, De Vos C. H. R, Lommen A, Hall R. D, Bino R. J, Van Der Plas L. H. W, Jansen R. C, Vreugdenhil D, Koornneef M (2006). The genetics of plant metabolism. Nat. Genet.

[77] Schauer N, Semel Y, Roessner U, Gur A, Balbo I, Carrari F, Pleban T, Perez-Melis A, Bruedigam C, Kopka J, Willmitzer L, Zamir D, Fernie A. R (2006). Comprehensive metabolic profiling and phenotyping of interspecific introgression lines for tomato improvement. Nat. Biotechnol.

[78] Lisec J, Meyer R. C, Steinfath M, Redestig H, Becher M, Witucka-Wall H, Fiehn O, Torjek O, Selbig J, Altmann T, Willmitzer L (2008). Identification of metabolic and biomass QTL in Arabidopsis thaliana in a parallel analysis of RIL and IL populations. Plant J.

[79] Keurentjes J.J.B, Sulpice R, Gibon Y, Steinhauser M C, Fu J, Koornneef M, Stitt M, Vreugdenhil D (2008). Integrative analyses of genetic variation in enzyme activities of primary carbohydrate metabolism reveal distinct modes of regulation in Arabidopsis thaliana. Genome Biol.

[80] Meyer R.C, Steinfath M, Lisec J, Becher M, Witucka-Wall H, Torjek O, Fiehn O, Eckardt A, Willmitzer L, Selbig J, Altmann T (2007). The metabolic signature related to high plant growth rate in Arabidopsis thaliana. Proc. Natl. Acad. Sci. USA.

[81] Wentzell A. M, Rowe H. C, Hansen B. G, Ticconi C, Halkier B. A, Kliebenstein D. J (2007). Linking metabolic QTLs with network and cis-eQTLs controlling biosynthetic pathways. PLoS Genet.

[82] Li Y, Breitling R, Jansen R. C (2008). Generalizing genetical genomics: getting added value from environmental perturbation. Trends Genet.

[83] Siva N (2008). 1000 Genomes project. Nat. Biotechnol.

[84] Ossowski S, Schneeberger K, Clark R. M, Lanz C, Warthmann N, Weigel D (2008). Sequencing of natural strains of Arabidopsis thaliana with short reads. Genome Res.

[85] Laubinger S, Zeller G, Henz S. R, Sachsenberg T, Widmer C. K, Naouar N, Vuylsteke M, Schölkopf B, Rätsch G, Weigel D (2008). At-TAX: A whole genome tiling array resource for developmental expression analysis and transcript identification in Arabidopsis thaliana. Genome Biol.

[86] Matsui A, Ishida J, Morosawa T, Mochizuki Y, Kaminuma E, Endo T. A, Okamoto M, Nambara E, Nakajima M, Kawashima M, Satou M, Kim J. M, Kobayashi N, Toyoda T, Shinozaki K, Seki M (2008). Arabidopsis transcriptome analysis under drought, cold, high-salinity and ABA treatment conditions using a tiling array. Plant Cell Physiol.

[87] Buckler E, Gore M (2007). An Arabidopsis haplotype map takes root. Nat. Genet.

[88] Sorkheh K, Malysheva-Otto L.V, Wirthensohn M.G, Tarkesh-Esfahani S, Martínez-Gómez P (2008). Linkage disequilibrium, genetic association mapping and gene localization in crop plants. Genet. Mol. Biol.

